# Isolation, culture, and delivery considerations for the use of mesenchymal stem cells in potential therapies for acute liver failure

**DOI:** 10.3389/fimmu.2023.1243220

**Published:** 2023-09-07

**Authors:** Hui Yang, Jiaxian Chen, Jun Li

**Affiliations:** State Key Laboratory for Diagnosis and Treatment of Infectious Diseases, National Clinical Research Center for Infectious Diseases, National Medical Center for Infectious Diseases, Collaborative Innovation Center for Diagnosis and Treatment of Infectious Diseases, The First Affiliated Hospital, Zhejiang University School of Medicine, Hangzhou, China

**Keywords:** mesenchymal stem cells (MSCs), acute liver failure (ALF), animal models, culture strategy, surface markers, cell therapy

## Abstract

Acute liver failure (ALF) is a high-mortality syndrome for which liver transplantation is considered the only effective treatment option. A shortage of donor organs, high costs and surgical complications associated with immune rejection constrain the therapeutic effects of liver transplantation. Recently, mesenchymal stem cell (MSC) therapy was recognized as an alternative strategy for liver transplantation. Bone marrow mesenchymal stem cells (BMSCs) have been used in clinical trials of several liver diseases due to their ease of acquisition, strong proliferation ability, multipotent differentiation, homing to the lesion site, low immunogenicity and anti-inflammatory and antifibrotic effects. In this review, we comprehensively summarized the harvest and culture expansion strategies for BMSCs, the development of animal models of ALF of different aetiologies, the critical mechanisms of BMSC therapy for ALF and the challenge of clinical application.

## 1 Introduction

Acute liver failure (ALF) is defined as a sudden onset of fulminant liver dysfunction in patients without underlying liver disease and is characterized by multiple organ failure with hepatic jaundice, coagulopathy [INR ≥ 1.5] and encephalopathy (1, 2). The interval time from the onset of jaundice to the development of encephalopathy is divided into three classifications: hyperacute, acute and subacute (3). Without therapeutic intervention, ALF can rapidly progress to multiorgan failure, severe systemic inflammation and even death, with a mortality rate often exceeding 90% (4). Liver transplantation, the only curative treatment for acute liver failure, is limited by the high cost, shortage of donor organs and long-term immune rejection (5). Hepatocyte transplantation has been considered an alternative to organ transplantation but has been hampered by the lack of large cell quantities, expansion difficulties *ex vivo*, rejection of allografts and xenotransplantation, and the rapid loss of liver properties *in vitro* (6–8). Therefore, other alternatives need to be studied for the constraint of hepatocyte and liver transplantation. Stem cells, including foetal biliary tree stem cells, foetal liver stem cells, haematopoietic stem cells, endothelial progenitor cells, MSCs, induced pluripotent stem cells and others, can transdifferentiate into hepatocyte-like cells to restore the damaged liver and response to stimulation (9). MSC therapy has been extensively studied and shows great clinical promise due to its ease of acquisition, strong proliferation ability, multipotential differentiation, homing to the lesion site, low immunogenicity and anti-inflammatory and antifibrotic effects.

**Figure 1:**
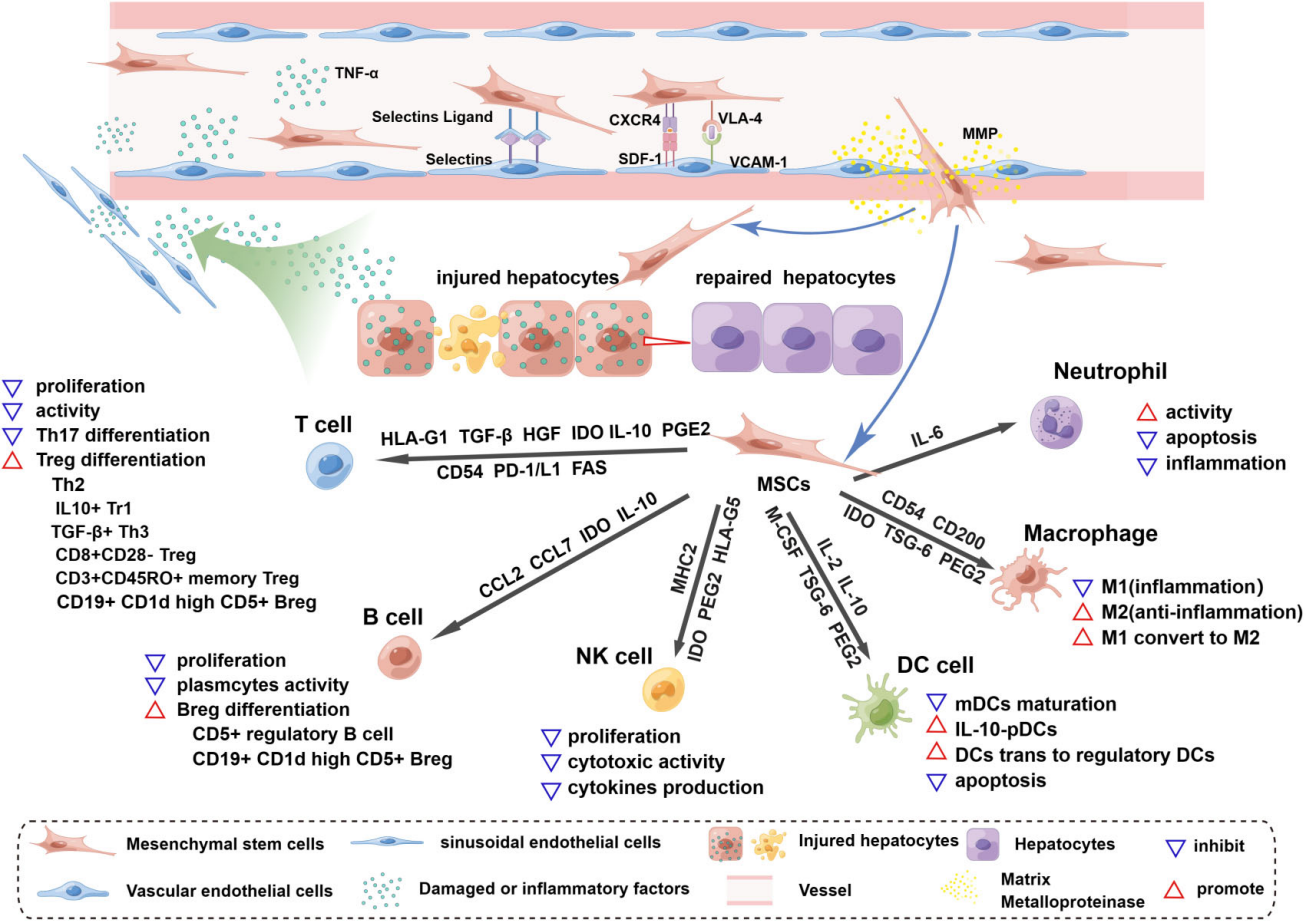
Homing and immunoregulation of MSCs triggered by the injured liver. The injured liver releases a variety of damaged or inflammatory factors, such as TNF-α and histamine, which enter vessels through the hepatic blood sinusoids and activate blood vascular endothelial cells (ECs), upregulating the expression of selectin and VCAM1. Once MSCs roll into the vessel wall, the expression of CD44 (HCAM), CXCR4, and VLA-4 on the surface of MSCs is triggered. MSCs then adhere to ECs through the interaction of selectin ligands and selectins, and an activation phase occurs through the interaction of SDF-1 and CXCR4, which enhances the affinity for integrins. The binding of VLA-4 and VCAM-1 promotes MSC extravasation; this process starts after the activation of matrix metalloproteinases (MMPs) and disrupts type IV collagen in the basement membrane. MSCs cross the basement membrane and are released into the hepatic interstitium, where they perform the functions of liver regeneration and immunoregulation and achieve therapeutic efficacy. MSCs release soluble factors and express surface molecules to regulate adaptive (T cells, B cells) and innate (NK cells, DC cells, macrophages and neutrophils) immune cells. HLA-G1, TGF-β, HGF and IDO secreted by MSCs can inhibit T-cell proliferation and activation, and similarly, the Fas and PD-1/L1 pathways trigger apoptosis of T cells. MSCs expressing CD54, PD-1, IL-10 and PEG2 can inhibit Th17 differentiation. In the presence of MSCs, T cells can differentiate into Tregs, such as Th2 cells, CD3^+^CD45RO^+^ memory Treg cells, CD8^+^CD28^-^ Tregs, IL10^+^ Tr1 cells and TGFβ^+^ Th3 cells. MSCs inhibit B-cell proliferation, interfere with the formation of plasma cells, and release CCL2 and CCL7 to inhibit antibody production. IDO from MSCs is involved in the proliferation of CD5^+^ regulatory B cells, and IL-10 promotes the differentiation of CD19^+^CD24^+^CD38^+^ Bregs. MSCs can inhibit the proliferation, cytotoxic activity and cytokine production of quiescent NK cells by releasing IDO, PGE2 and HLA-G5 and by expressing MHCI. The maturation of myeloid dendritic cells (mDCs) is inhibited by MSC-derived IL-6, macrophage colony-stimulating factor (M-CSF), TSG-6, and PGE2. IL-10-plasmacytoid dendritic cells (pDCs) can be induced by PGE2 release from MSCs. In addition, MSCs can induce the transformation of mature dendritic cells into immunosuppressive regulatory dendritic cells through IL-10 and evasion of apoptosis. Inflammatory macrophages (M1) are converted to anti-inflammatory macrophages (M2) by IDO, TSG-6 and PGE2 secreted by MSCs, and CD54 and CD200 enhance their immunosuppressive effects. MSCs maintain neutrophil activity, and IL-6 delays apoptosis and inflammation.

At present, MSCs are utilized to improve liver function while waiting for liver transplantation and can also be used as a potential alternative therapy to organ or hepatocyte transplantation (10). Some recent clinical trials have reported that infusion of MSCs could induce tolerance after liver transplantation to reduce immune rejection due to the low immunogenicity and immunosuppression of MSCs (11–13). MSCs have been isolated from multiple biological tissues, including adult bone marrow, adipose tissue and neonatal tissues, such as the umbilical cord and the placenta. Bone marrow-derived mesenchymal stem cells (BMSCs) were the first multipotential stromal progenitor cells isolated and identified. BMSCs was recognized as the most promising cell sources due to their easy access and well-characterized biological features in clinical trials and preclinical studies (14, 15). However, only a small percentage of BMSCs, ranging from 0.01% to 0.001%, are present in bone marrow tissue, and these cells require substantial expansion *ex vivo* before they can be used for clinical treatment. Isolating BMSCs through conventional differential adherence and density gradient centrifugation is effective, but the approaches do not yield a relatively homogeneous cell population, and the cells may be contaminated by other cells from the bone marrow (16), which results in differential proliferation, transdifferentiation and therapeutic efficiency of BMSCs. Therefore, cell sorting based on BMSC-specific markers is an attractive technique for homogeneous subsets, and glucose, hypoxia and serum-free conditions are vital to facilitate the proliferation of MSCs and reduce cell senescence. Although BMSCs have been used in numerous clinical therapies and animal investigations (17, 18), the mechanism is unclear. In this review, we summarize the critical mechanism as shown in **Figure 1**. Under the stimulation of liver failure signals, BMSCs homed to lesion sites through the endothelium and immediately modulated the immune microenvironment, which is beneficial for tissue repair. Furthermore, several animal models of ALF have been described to clarify the mechanisms of BMSCs for the treatment of ALF with different aetiologies.

## 2 Definition and source of mesenchymal stem cells

**Figure 2:**
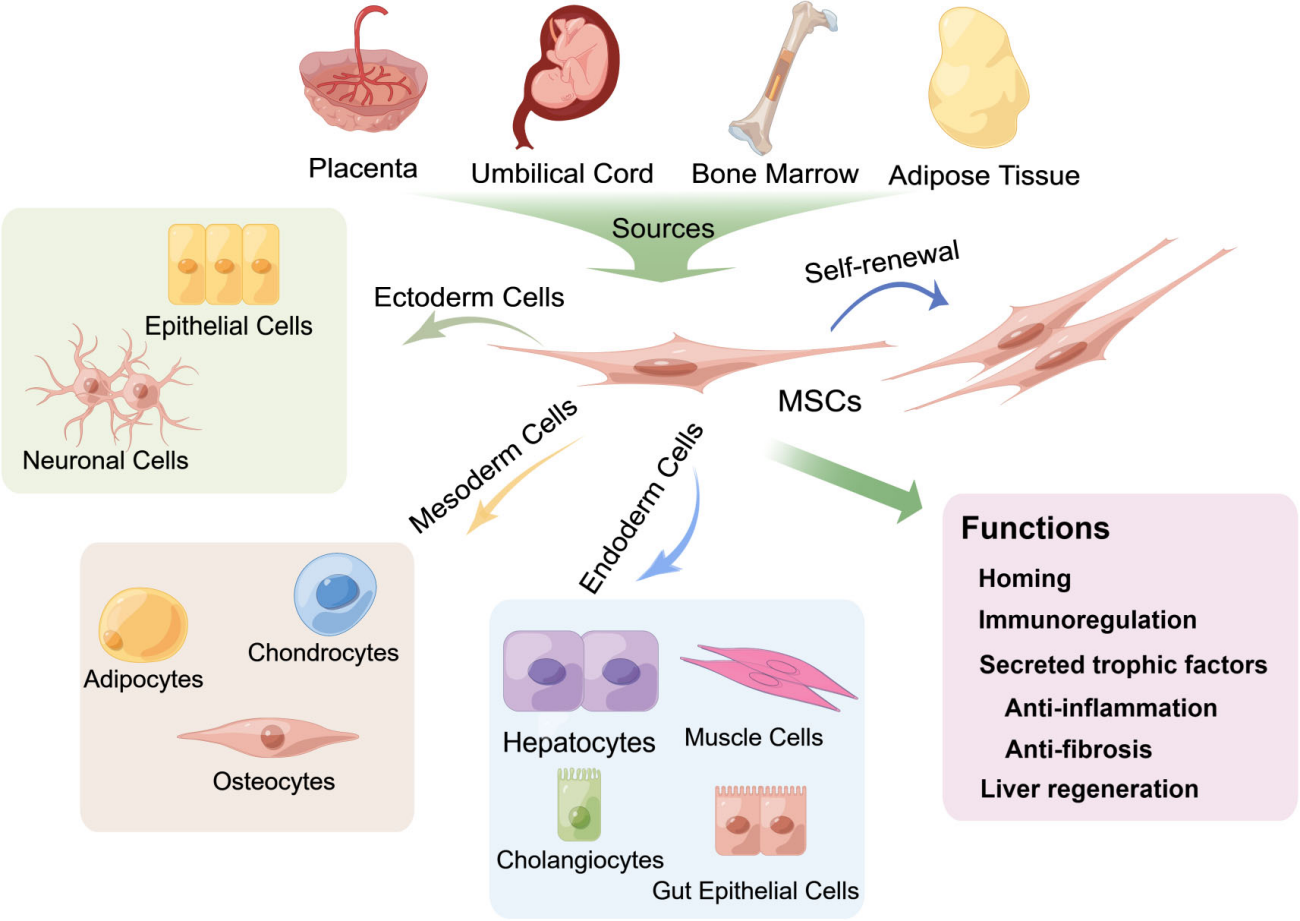
Multipotent differentiation and functions of mesenchymal stem cells (MSCs) derived from various tissues. MSCs can be isolated from multiple biological tissues, including adult bone marrow, adipose tissue and neonatal tissues, such as the umbilical cord and the placenta, and can differentiate into osteocytes, chondrocytes, adipocytes and other mesodermal lineages (21) and exhibit endodermic (such as hepatocytes, muscle cells, gut epithelial cells, and cholangiocytes) (20, 22–28) and ectodermic (epithelial cells and neuronal cells) (29–33) differentiation potential. MSCs also have strong self-renewal properties. The fundamental biological functions of MSCs include homing to sites of damage, immunoregulation of the immune microenvironment and secretion of trophic factors that exert anti-inflammatory and antifibrotic effects, as well as differentiation into hepatocytes to promote liver regeneration.

MSCs are a heterogeneous population that can adhere to plastic and proliferate *ex vivo*, forming colonies with a fibroblast-like morphology. They can differentiate into osteocytes, chondrocytes, adipocytes and other mesodermal lineages and have endodermic (19) and ectodermic (20) differentiation potential (**Figure 2**). Several studies have shown that MSCs can differentiate into functional hepatocytes and cholangiocytes after growth factor induction *ex vivo* (22). Intrasplenic transplantation of human-derived BMSCs into mice with fulminant liver failure developed a dual humanized mouse model with hepatocytes and immune cells (23). A recent investigation reported that MSCs can also self-assemble a three-dimensional (3D) human liver bud *ex vivo* by transdifferentiating into hepatocytes, sinusoidal endothelial cells (LECs) and hepatic stellate cells (HSCs) (24). Minimal criteria for human MSCs in basic scientific investigations and preclinical studies were proposed in 2006 by the International Society for Cellular Therapy (ISCT), which included adherence to plastic, potential for differentiation into osteoblasts, adipocytes, and chondroblasts under standard in *ex vivo* differentiation conditions, and expression (≥95% positive) of CD105, CD73 and CD90, as measured by flow cytometry. Additionally, these cells must lack expression (≤2% positive) of CD45, CD34, CD14 or CD11b, CD79a, CD19 and HLA class II, which are haematopoietic stem cell surface antigens (21).

MSCs are located in multiple adult and neonatal tissues with perivascular niches (34), such as adult bone marrow and adipose tissue, and neonatal tissues, such as the umbilical cord and the placenta. In addition, the fundamental biological functions of MSCs involved in the treatment of liver diseases are mainly homing/migration to sites of damage and the secretion of trophic factors that mediate liver regeneration and regulation of immune responses (17, 35–37) (**Figure 2**).

## 3 Culture strategies of BMSCs *in vitro*

### 3.1 Isolation of BMSCs

The effect of BMSCs in clinical treatment is highly dependent on their quality. However, the lack of standardized culture procedures and unique markers limits the consistency of BMSC characteristics. The initial step for BMSC standardization is the isolation process (38). Human bone marrow is mainly obtained from the iliac crest via aspiration in the presence of some anticoagulants, such as heparin sodium (23). There are several methods for isolating BMSCs from bone marrow. Traditional differential adhesion is based on the typical capacity of MSCs, such as adherence to plastic and sensitivity to enzyme digestion, and the culture medium is changed every 3-4 days to gradually achieve purification. Although this method is convenient and economical, it does not yield a homogeneous population of cells that contain other bone marrow subpopulations, such as endothelial cells, pericytes, leukocytes, and haematopoietic stem cells (39, 40). Another technique, called density gradient centrifugation, has also been proposed to precipitate different cells in the bone marrow according to size and density using gradient centrifugation solutions with a density of approximately 1.077 g/mL, low viscosity and low permeability, such as Ficoll, Ficoll-Paque, Percoll, and Lymphogre. After centrifugation and stratification, the greyish-white cloudy layer above the separation fluid was purified through differential adhesion. However, the lack of specific subsets and contamination of cell populations have limited its application (38, 41, 42). To improve the homogeneity of BMSC populations, advanced isolation methods, such as fluorescence-activated cell sorting (FACS) and magnetic-activated cell sorting (MACS), have been used for high-throughput screening of BMSCs via specific surface markers. FACS and MACS employ electric and magnetic fields, respectively, that exert external forces to separate BMSCs. However, there is no evidence assessing their influence on MSC functions and therapeutic effects (43, 44). Therefore, the crucial aspect of this method is specific surface markers. ISCT published minimal guidelines for the isolation of human BMSCs based on the positive markers CD105, CD73, and CD90 and the negative markers CD45, CD34, HLA-DR, CD79a, CD19, CD11b, and CD14 (21), and we summarized a variety of unique markers for human BMSCs in **Table 1** (45–58).

**Table 1:** Positive and negative markers of human bone marrow mesenchymal stem cells.

Gene	Gene Symbol	ENSG	Description	Expression
CD29	ITGB1	ENSG00000150093	Integrin Subunit Beta 1	+
CD44	CD44	ENSG00000026508	CD44 Molecule (Indian Blood Group)	+
CD49d	ITGA4	ENSG00000115232	Integrin Subunit Alpha 4	+
CD49e	ITGA5	ENSG00000161638	Integrin Subunit Alpha 5	+
CD51	ITGAV	ENSG00000138448	Integrin Subunit Alpha V	+
CD54	ICAM1	ENSG00000090339	Intercellular Adhesion Molecule 1	+
CD71	TFRC	ENSG00000072274	Transferrin Receptor	+
CD73	NT5E	ENSG00000135318	5’-Nucleotidase Ecto	+
CD90	THY1	ENSG00000154096	Thy-1 Cell Surface Antigen	+
CD105	ENG	ENSG00000106991	Endoglin	+
CD120a	TNFRSF1A	ENSG00000067182	TNF Receptor Superfamily Member 1A	+
CD120b	TNFRSF1B	ENSG00000028137	TNF Receptor Superfamily Member 1B	+
CD146	MCAM	ENSG00000076706	Melanoma Cell Adhesion Molecule	+
CD166	ALCAM	ENSG00000170017	Activated Leukocyte Cell Adhesion Molecule	+
CD271	NGFR	ENSG00000064300	Nerve Growth Factor Receptor	+
CD124	IL4R	ENSG00000077238	Interleukin 4 Receptor	+
CD49f	ITGA6	ENSG00000091409	Integrin Subunit Alpha 6	+
CD49a	ITGA1	ENSG00000213949	Integrin Subunit Alpha 1	+
CD49b	ITGA2	ENSG00000164171	Integrin Subunit Alpha 2	+
CD49c	ITGA3	ENSG00000005884	Integrin Subunit Alpha 3	+
CD58	CD58	ENSG00000116815	CD58 Molecule	+
CD61	ITGB3	ENSG00000259207	Integrin Subunit Beta 3	+
CD200	CD200	ENSG00000091972	CD200 Molecule	+
CD102	ICAM2	ENSG00000108622	Intercellular Adhesion Molecule 2	+
CD104	ITGB4	ENSG00000132470	Integrin Subunit Beta 4	+
CD221	IGF1R	ENSG00000140443	Insulin Like Growth Factor 1 Receptor	+
CD140a	PDGFRA	ENSG00000134853	Platelet Derived Growth Factor Receptor Alpha	+
CD140b	PDGFRB	ENSG00000113721	Platelet Derived Growth Factor Receptor Beta	+
PODXL	PODXL	ENSG00000128567	Podocalyxin Like	+
SOX11	SOX11	ENSG00000176887	SRY-Box Transcription Factor 11	+
SSEA3	B3GALT5	ENSG00000183778	Beta-1,3-galactosyltransferase 5	+
H-L6	TM4SF1	ENSG00000169908	Transmembrane 4 L six family member 1	+
GD2	B4GALNT1	ENSG00000135454	Beta-1,4-N-acetyl-galactosaminyltransferase 1	+
MSCA-1	ALPL	ENSG00000162551	Alkaline phosphatase, biomineralization associated	+
SSEA-4	ST3GAL2	ENSG00000157350	ST3 beta-galactoside alpha-2,3-sialyltransferase 2	+
Stro-1			Record to support submission of GeneRIFs for a gene not in Gene (human)	+
CD13	ANPEP	ENSG00000166825	Alanyl Aminopeptidase, Membrane	+
CD106	VCAM1	ENSG00000162692	Vascular Cell Adhesion Molecule 1	+
CD56	NCAM1	ENSG00000149294	Neural Cell Adhesion Molecule 1	+/━
CD309	KDR	ENSG00000128052	Kinase Insert Domain Receptor	+
Nucleostemin	GNL3	ENSG00000163938	G Protein Nucleolar 3	+
NRP1	CD304	ENSG00000099250	Neuropilin 1	+
CD81	CD81	ENSG00000110651	CD81 Molecule	+/━
CD130	IL6ST	ENSG00000134352	Interleukin 6 Cytokine Family Signal Transducer	+
SUSD2/W5C5	SUSD2	ENSG00000099994	Sushi Domain Containing 2	+/━
NG2	CSPG4	ENSG00000173546	Chondroitin Sulfate Proteoglycan 4	+
Nestin	NES	ENSG00000132688	Nestin	+
CD11a	ITGAL	ENSG00000005844	Integrin Subunit Alpha L	━
CD11b	ITGAM	ENSG00000169896	Integrin Subunit Alpha M	━
CD14	CD14	ENSG00000170458	CD14 Molecule	━
CD19	CD19	ENSG00000177455	CD19 Molecule	━
CD31	PECAM1	ENSG00000261371	Platelet And Endothelial Cell Adhesion Molecule 1	━
CD34	CD34	ENSG00000174059	CD34 Molecule	━
CD45	PTPRC	ENSG00000081237	Protein Tyrosine Phosphatase Receptor Type C	━
CD117	KIT	ENSG00000157404	KIT Proto-Oncogene, Receptor Tyrosine Kinase	━
CD79A	CD79A	ENSG00000105369	CD79a Molecule	━
HLA Class II				━

+ represents a positive marker of human bone marrow mesenchymal stem cells; ━ represents a negative marker of human bone marrow mesenchymal stem cells; and +/━ indicates that it is not clear whether the marker is a positive or negative marker.

### 3.2 Culture expansion of BMSCs

The scientific studies and clinical applications of BMSCs require substantial expansion *ex vivo* to obtain sufficient numbers of cells because MSCs are rare in the bone marrow (0.001-0.01% of total nucleated cells and 0.42% of plastic adherent cells) (19). To maintain the functions and activities of BMSCs *in vitro*, a variety of approaches have been used to optimize the culture conditions, including medium composition, cell seeding density, passages and parameters related to the external environment, such as oxygen tension, pH and temperature.

#### 3.2.1 Serum

Foetal bovine serum (FBS), the classic nutritional supplement for cell culture *ex vivo*, has been commonly used at a concentration of 5-20% (v/v) for the expansion of BMSCs, predominantly at 10% (59). FBS can provide macromolecules, proteins, adhesion, growth factors, nutrients, hormones and other essential biomolecules for the growth of BMSCs (60). However, the treatment of BMSCs cultured with FBS is controversial due to high batch-to-batch variations, xenoimmune effects and contamination with pathogens (38, 61, 62). Therefore, materials from autologous or allogeneic human blood sources have been explored, and the results showed that human serum (63), platelet lysate (63, 64) and umbilical cord serum (65) significantly increased cell proliferation, but an in-depth study of their efficacy is lacking. To overcome the uncertainties associated with serum, commercial serum-free media (SFMs) showed good performance *ex vivo* culture of human-derived BMSCs (63, 66–68). Van T Hoang et al. adapted a standardized process to assess the functional characteristics of serum-free cultured MSCs and showed that the MSCs satisfied the criteria (including basic MSC characteristics, normal karyotype, stronger proliferation, clinical-scale production and quality control requirements) (69, 70).

#### 3.2.2 Glucose

Glucose is another critical source of energy for the growth and development of most cell types *in vivo (* 71). Under physiological conditions, the serum glucose concentration of an organism is maintained at approximately 100 mg/dL, suggesting that MSCs should be exposed to the same glucose concentration in the bone marrow niche or *ex vivo* culture (72). Nevertheless, there is still controversy regarding the culture of BMSCs in terms of high vs. low glycaemic levels. Al-Qarakhli et al. assessed the effects of different glucose concentrations on the proliferation, senescence and multidirectional differentiation ability of MSCs, and the results demonstrated that high concentrations of glucose (450 mg/dL) inhibited osteogenic/adipogenic differentiation and had a limited negative effect on the proliferation and stemness of MSCs (73). Similar studies have shown that low glucose concentrations (100 mg/dL or 350 mg/dL) during culture can promote cell proliferation, colony formation, and multidirectional differentiation and reduce apoptosis and senescence (74–78). Overall, low-glycaemic culture may better maintain the properties of MSCs, which may facilitate the homing and tissue repair of MSCs in ALF.

#### 3.2.3 Oxygen tension

The oxygen tension (pO_2_) of MSCs exposed to the bone marrow microenvironment typically ranges from 1% to 8% (also referred to hypoxia) (79), whereas *ex vivo* culture was at an atmospheric oxygen tension (21%), which may lead to cell proliferation cessation after multiple passages as well as cellular senescence. This does not occur in hypoxic conditions (1% pO_2_), which is possibly related to downregulation of the gene expression of p16 and extracellular signal-regulated kinase (ERK) (80). Several previous studies have revealed that hypoxic culture can promote cell proliferation (81), inhibit differentiation (82, 83) and reduce BMSC senescence (80). Recently, Ben Antebi et al. evaluated the function of human and porcine bone marrow-derived MSCs following long-term (10 days) and short-term (48 hours) hypoxic (1% pO2) culture, and the results demonstrated that short-term culture under hypoxia significantly increased cell proliferation upregulated VEGF expression and downregulated the expression of HMGB1 and the apoptotic genes BCL-2 and CASP3. Additionally, in short-term hypoxic culture at 2% and 5% pO2, BMSCs showed inhibition of the proinflammatory cytokine IL-8 and promotion of the anti-inflammatory agents IL-1Ra and GM-CSF, especially in short-term hypoxic culture at 2% pO2 (84). Yu et al. found that CXCR4 expression was upregulated in the presence of short-term hypoxic culture (24 h, 2% pO2) and low-dose inflammatory stimuli (1 ng/mL TNF-α and 0.5 ng/mL IL-1β), enhancing the homing/migration of BMSCs (85).

#### 3.2.4 Cell seeding density

Cell seeding density is a key factor to be considered when BMSCs are expanded *ex vivo*. During the primary culture of bone marrow cell suspensions, the seeding density is typically 1 × 10^6^ to 2 × 10^6^ cells/cm^2^ for differential adhesion and approximately 1 × 10^4^ cells/cm^2^ for density gradient centrifugation (86). The inoculation density of BMSCs is usually in the range of 2,000 to 5,000 cells/cm^2^ during the passaging process (38). It has been shown that low-density inoculation may facilitate cell proliferation by reducing contact inhibition and less affect the cell surface antigen phenotype and cell differentiation.

#### 3.2.5 BMSC passage

Passaging is helpful to expanding the number of cells in culture and avoids mass mortality arising from cells entering the plateau stay or even decay. Typical digestion with 0.25% trypsin/EDTA was performed on cells at a confluence of 80-90%. BMSCs face replicative senescence, exhibiting progressive shortening of chromosomal telomeres, reduced stemness and a heightened risk of mutation (87, 88). Although no comprehensive studies have reported which generation will undergo cell senescence, the loss of typical fibroblast-like morphology and the decreased rate of fibroblast colony-forming units (CFU) are representations of *ex vivo* cellular ageing (88–90). An *ex vivo* study also revealed a dramatic decrease in the potential for hepatic differentiation at later passages (passage 8, p8) (91), suggesting that early passage of BMSCs may have superior therapeutic benefits for liver failure.

## 4 Homing functions of MSCs in ALF treatment

Homing to damaged tissue sites is a key property of BMSCs in treating liver failure. Regardless of the method of local or systemic administration, BMSCs are always found at sites of damaged tissue (92). Proinflammatory chemokines, such as TNF-α and histamine secreted by the injured liver, activate blood vascular endothelial cells (ECs), as indicated by the upregulated expression of selectin and VCAM1 (93). Once MSCs rolled to the vascular wall, a few significant ligands related to MSC extravasation, such as CD44 (HCAM), CXCR4, and VLA-4, were triggered. The adherence process occurs when the adhesion molecule selectin ligand CD44 (HCAM) expressed by MSCs interacts with selectins located on ECs (94, 95). Notably, CD44 is a significant target for modifying MSCs. During the activated phase, stromal cell-derived factor (SDF)-1 naturally expressed by ECs binds to the chemokine receptor CXCR4 on MSCs or CXCR7 and other chemokines, such as MCP-1 and MCP-3 (96–99), increasing the affinity for integrins (100). Several preclinical studies indicated that MSCs with enhanced CXCR4 expression after genetic engineering, treatment with ALF rat serum or stimulation with inflammatory cytokines such as TNF-α and IL-1β/3 showed better homing *in vivo* (101–106). The entrapment phase involves the VLA-4-VCAM-1 interaction, which is the key mediator of the MSC adherence process to ECs (107, 108). During the extravasation stage, the transmigration of MSCs begins with the activation of matrix metalloproteinases (MMPs), which break down type IV collagen in the basement membrane and then cross the endothelial cell layer and basement membrane into the vasculature to migrate to the liver lesion (109, 110), where they can then exert their therapeutic, regeneration and immunomodulatory effects.

## 5 Immunomodulatory functions of MSCs in ALF treatment

MSCs can improve and repair injured tissue by regulating immune responses by secreting soluble factors and direct cell-to-cell interactions (111). When MSCs migrate to damaged sites, they interact closely with numerous proinflammatory cytokines, such as TNF-α, IL-1β and IL-6, causing the conversion of MSCs to an immunosuppressive phenotype to modulate innate and adaptive immune responses (112). In this section, we mainly focus on soluble factors and membrane-bound molecules involved in MSC immune modulation (**Figure 1**).

### 5.1 Adaptive immune cells

MCSs can inhibit T‐cell proliferation and activation and induce the differentiation of Tregs. Several soluble immunosuppressive factors secreted by MSCs are involved in T-cell immunoregulation; the release of HLA-G1, TGF-β and HGF induces cell cycle arrest in G1 phase by downregulating phosphoretinoblastoma (pRb), cyclin D and cyclin A as well as upregulating cyclin-dependent kinase inhibitor 1B (p27Kip1), rendering T-cell activation ineffective (113, 114), and the production of IDO after IFN‐γ stimulation promotes tryptophan metabolism, resulting in the depletion of tryptophan, which inhibits proliferation and induces apoptosis of T cells (115). The direct interaction between MSCs and T cells may trigger T-cell apoptosis through the Fas ligand (FasL)-dependent pathway (116) as well as the PD-L1 pathway (117); of note, Fas ligand-associated T-cell apoptosis can induce macrophages to produce TGF-β, thereby increasing the abundance of Tregs (116). Additionally, several studies revealed that MSCs could inhibit the differentiation of Th17 cells through different mechanisms; the expression of CD54 recruited Th17 cells to MSCs and then upregulated PD‐1, IL‐10 and PGE2, blocking differentiation (118, 119). Some reports have also shown that activated T cells can differentiate into Th2 (120), CD3^+^CD45RO^+^ memory Treg cells (121), CD8^+^CD28^‐^ Treg (122), IL10^+^ Tr1 and TGFβ^+^ Th3 (123) cells in the presence of MSCs, which suppress immune responses and accelerate tissue repair. Although the detailed mechanism of the interaction between MSCs and B cells is controversial, it is known that the inhibition of B-cell proliferation by MSCs seems to be associated with cell cycle disruption at specific stages rather than the induction of apoptosis (124, 125). Activated MSCs can interfere with the formation of plasmacytes and promote the differentiation of Bregs; for example, the soluble molecule IDO secreted by MSCs is involved in the survival and proliferation of CD5^+^ regulatory B cells (126), and when MSCs express IL-10, they promote the production of CD19^+^CD24^+^CD38^+^ Bregs in humans and CD19^+^CD1d high CD5^+^ Bregs in mice (126, 127). In addition, MSCs can promote the formation of naive, transitional and memory B-cell subsets, and these nonactivated B cells can induce Treg differentiation. Notably, the MSC-derived CC chemokine ligands CCL2 and CCL7 can suppress immunoglobulin (such as IgA and IgM IgG) production and release by plasmacytes (128).

### 5.2 Innate immune cells

Natural killer cells (NK cells) are important effector cells of innate immunity (129). MSCs can inhibit the proliferation, cytotoxic activity and cytokine production of resting NK cells (130). IL-2-mediated proliferation of resting NK cells is inhibited by coculture with MSCs. The mechanism may involve the release of IDO, PGE2 and HLA-G5 (131–133), and upregulating the expression of HLA class I molecules (MHCI) inhibits cytokine-mediated induced NK-cell cytotoxicity and decreases the secretion of cytokines (130). MSCs also maintain the activity of neutrophils for a long period to promote the elimination of invading bacteria (134), and the MSC-derived soluble factor IL-6 can delay apoptosis of neutrophils and inhibit the neutrophil-mediated fulminant inflammatory response, which is called the respiratory burst (135). The immunoregulatory effect of MSCs on macrophages mainly converts polarized inflammatory macrophages (M1) to anti-inflammatory macrophages (M2). The mechanism may involve IDO, TSG-6 and PGE2 (134, 136–138). Furthermore, proinflammatory factors (IFN‐γ, TNF‐α and LPS) can enhance the M2 macrophage polarization of MSCs. To date, the membrane-bound molecules CD54 and CD200 have been found to increase the immunosuppressive function of MSCs (139, 140). Regarding myeloid dendritic cells (mDCs), MSCs can inhibit the development and maturation of mesenchymal/dermal DCs and the conversion of umbilical cord blood and CD34^+^ haematopoietic progenitors as well as monocytes into DCs (141) (142, 143). Several recent studies suggested that MSC-derived IL-6, macrophage colony-stimulating factor (M-CSF), TSG-6 and PGE2 could be responsible for the immunoregulatory interaction between MSCs and immature dendritic cells (141, 142, 144). Furthermore, MSCs can induce the transformation of mature dendritic cells into immunosuppressive regulatory dendritic cells via jagged-2 and IL-10-activated SOCS3 pathways while escaping from their apoptotic fate (145, 146), and IL-10-plasmacytoid dendritic cells (pDCs) can be induced by PGE2 (124).

These soluble and membrane molecules play important roles in MSC immunomodulation, and researchers can evaluate the effectiveness of pretreatment *in vitro* for improving the immunomodulation of MSCs. Recently, the concept of immune training of MSCs has been proposed (147–149), where MSCs are stimulated *in vitro* by proinflammatory factors or cocultured with activated immune cells, and when MSCs are stimulated again with the same stimuli, detection of the immunosuppressive molecules we mentioned can be used to assess whether MSCs can achieve “memory” to rapidly and efficiently suppress inflammatory signaling.

## 6 Developing animal models of liver failure and the mechanism of MSC therapy

### 6.1 Devascularization-induced ischaemia-reperfusion model

Devascularization models mainly imitate hepatic ischaemia-reperfusion injury (IRI) caused by liver transplantation, hepatectomy and haemorrhagic shock (150, 151), and such models have also been used to investigate liver regeneration and the therapeutic potential of artificial liver support systems (ALSSs) (152, 153). Hepatocytes and endothelial cells experienced hypoxic insult during a brief period of ischaemia. Subsequently, dysfunctional mitochondrial respiratory chain-activating degradative enzymes cause a range of disruptions in intracellular proteins, lipids and DNA. Reperfusion produces reactive oxygen species (ROS) and hydroxyl radicals that activate Kupffer cell amplification cascades and inflammatory responses, recruit neutrophils (154), and trigger different types of cell death, including apoptosis, autophagy-associated cell death and necrosis (155).

As many investigations have shown, ischaemia-reperfusion models in rats (156), mice (157) (C57BL/6 mice were described as the most popular model) and pigs (158) (in combination with hepatectomy) have been developed via 70% hepatic segmental thermal ischaemia. The critical protocol is a noninvasive vascular clip on the upper left side of the portal triad structure (bile duct, portal vein and hepatic artery) for 45 (159), 60 or 90 (160) minutes to block the blood supply to the left and median lobe of the liver, and reperfusion is initiated by removal of the clamp (161). A previous study revealed reproducible hepatic injury at 60 min of ischaemia and is therefore extensively employed in ischaemia-reperfusion models (157, 161–164), allowing decompression of the portal vein through the right lobe and caudate lobe. To prevent mesenteric vein congestion, all surgical procedures were performed at a constant temperature of 37°C.

Tail vein (165, 166), hepatic vein (167) and peripheral vein (168) injection of MSCs in an ischaemia-reperfusion injury animal model showed that MSC infusion can reduce liver damage and cell death (165, 167), improve the levels of ALT and AST (166–168) and mainly decrease oxidative stress caused by liver excision and ischaemia-reperfusion injury (169). MSCs also inhibit the production of proinflammatory cytokines (TNF-α, IL-1β and iNOS), macrophage activation and neutrophil recruitment and promote anti-inflammatory cytokine secretion (IL-10), which is beneficial for recovery from liver injury and inflammatory responses (167). The potential mechanism of MSCs in the treatment of liver IRI may increase CD47 expression in the liver, and then the CD47-SIRPα interaction activates HEDGEHOG/SMO/Gli1 signaling and further inhibits NEK7/NLRP3 activity to protect the integrity of the liver (166). Zheng et al. found that MSCs upregulated PINK1-dependent mitophagy and exerted a protective effect in liver IRI, which might be associated with the modulation of AMPKα activation (168).

### 6.2 Acetaminophen-induced drug-induced liver injury models

Acetaminophen-induced drug-induced liver injury (DILI) is the most frequent cause of ALF in many Western countries, such as the United States and the United Kingdom. The animal models caused by acetaminophen are more similar to the pathophysiological characteristics of liver failure in humans (170). The toxicity mechanism of excess acetaminophen-induced oncotic necrosis begins with the accumulation of a toxic metabolite, N-acetyl-benzoquinone imine (NAPQI), catalysed by the cytochrome P450 enzyme system (171). Subsequently, a large amount of reactive oxygen species are formed, which initiates severe mitochondrial oxidative stress (172), mainly through activation of MAP kinase and translocation of phospho-c Jun N-terminal kinase (p-JNK) to mitochondria (173); the mitochondria eventually undergo swelling of the cytoplasm and rupture of the outer membranes (174), with the release of endonucleases to degrade nuclear DNA (175, 176). Several species, such as mice, rats, rabbits, dogs and pigs, have been used to develop acetaminophen models, and mice are widely used because they develop liver failure very close to those reported in humans in pathophysiology structure and acetaminophen doses (177).

Furthermore, the acetaminophen dose, the diluent, the administration route and the mouse strain are critical factors that need to be considered for model development. Many studies have noted that because glutathione can relieve the toxicity associated with NAPQI (178), fasting before acetaminophen administration maintains baseline levels of glutathione in all animals, which increases the consistency of experimental results and the success of acetaminophen-induced liver failure. In previous studies, typical hepatotoxicity was observed in fasted mice at doses of 200-300 mg/kg and in nonfasted mice at doses of 500-600 mg/kg or higher, and acetaminophen was diluted in normal saline (NS) or phosphate-buffered saline (PBS) and administered intraperitoneally to mice, while intravenous or subcutaneous administration is more suitable for large animals.

Some studies have reported that tail vein transplantation of MSCs significantly improves the survival rate of mice with liver failure induced by APAP and ameliorates liver function by reducing intense centrilobular necrosis and inflammatory infiltration (179–183). A recent study showed that MSC therapy can efficiently improve APAP-induced mitochondrial dysfunction and liver injury by inhibiting c-Jun N-terminal kinase (JNK)-mediated mitochondrial retrograde pathways (179). Another study reported that MSC-mediated immunoregulation is associated with the activation of the Notch2/COX2/AMPK/SIRT1 pathway (183). More interestingly, MSCs can enhance antioxidant activity to attenuate liver damage by inhibiting cytochrome P450 activity (by reducing NAPQI production) to reduce the depletion of GSH and oxidative stress. These results might be related to the downregulation of MAPK signalling and the decreased inflammatory responses (180).

### 6.3 Carbon tetrachloride-induced drug-induced liver injury model

Carbon tetrachloride (CCL4), a classical hepatotoxin, induces DILI after single high-dose administration and can progress to chronic liver disease (CLD) (184) such as nonalcoholic steatohepatitis (NASH) (185), hepatocellular carcinoma (HCC) (186), acute-on-chronic liver failure or other alcohol-related liver disease and fatty liver disease after multiple low-dose administrations (187). After entering the body, CCL4 depends on the cytochrome P450 enzyme system metabolism for conversion into reactive trichloromethyl radicals with high activity (188). These metabolites can cause lipid peroxidation and hepatocyte membrane rupture, as well as DNA strand breakage. In addition, it has been revealed that such damage further affects the transcriptional and replication activity of hepatocytes, resulting in portacaval zone necrosis (189).

In general, CCL4 administration is performed in mice, rats and rabbits (BALB/c mice have been described as the most appropriate model) via intragastric administration, intraperitoneal injection, subcutaneous injection or inhalation to induce acute or chronic liver failure. In some investigations, 6- to 8-week-old male mice (weighing approximately 25-30 g) were used to develop acute mouse models of CCL4 induction (190, 191), in which olive or corn oil served as diluents to solubilize CCL4 at ratios ranging from 10% (v/v) to 50% (v/v), and CCL4 doses of 2 mL/kg or higher can induce acute liver failure in mice (191–194).

MSC therapy efficiently prolonged the survival time of CCL4 induced acute liver failure mice from day 2 to day 7 after transplantations of second trimester amniotic fluid (AF-MSCs), and the ALT and AST levels significantly decreased by 35.36% and 64.72%, respectively (195). In addition, Milosavljevic et al. found that MSCs can modulate the IL17 signaling to treat the immune-mediated liver failure via altering NKT17/NKTreg ratio and suppressing hepatotoxicity of NKT cells in an IDO-dependent manner (196). A previous study reported that the treatment effect of adipose tissue-derived mesenchymal stem cells (AT-MSCs) may relate to the secretion of interleukin 1 receptor (IL-1R), IL-6, IL-8, granulocyte colony-stimulating factor (G-CSF), granulocyte-macrophage colony-stimulating factor (GM-CSF), monocyte chemotactic protein 1, nerve growth factor, and hepatocyte growth factor (197).

### 6.4 D-gel/LPS-induced TNF-α-mediated liver failure model

Lipopolysaccharide (LPS) is a molecule that is present in the outer membrane of gram-negative bacteria and can activate Kupffer cells (198–200), triggering the secretion of multiple inflammatory mediators (200, 201), and the coadministration of 300 mg/kg D-Gal dramatically increased rodent susceptibility to LPS, resulting in extensive liver injury and cell death (202). D-Gal is a hepatocellular phosphate uracil nucleotide interference agent that is metabolized via the galactose pathway and can cause diffuse necrosis and inflammation rather than zonal necrosis, similar to most hepatotoxic drugs (153). The administration of D-Gal/LPS in mice induced liver necrosis and inflammation similar to human hepatitis (203, 204). Previous studies demonstrated that coadministration of LPS at doses of 10-100 µg/kg and D-Gal at doses of 100-1000 mg/kg was performed to establish an acute liver failure mouse model (205–209).

The pathophysiological mechanism of D-Gal/LPS-induced ALF involves the binding of LPS to Toll-like receptor 4 (TLR4) on Kupffer cells, which triggers transcriptional and translational activation of cytokines such as TNF-α, IL-1β and IL-6 (210, 211). In particular, TNF-α has been recognized as a key regulator of hepatitis, as it recruits many neutrophils into the liver sinusoids and induces the expression of various adhesion molecules, including intercellular adhesion molecule 1 (ICAM1), vascular adhesion molecule 1 (VCAM1), selectin, and chemokines on endothelial cells and hepatocytes, after LPS treatment (212, 213). Some of these adhesion molecules are critical for neutrophil extravasation and cytotoxicity. In addition, by binding to its receptor 1 (TNFR1) on hepatocytes, TNF-α activates the nuclear factor kappa beta (NF-κB) pathway, resulting in the expression of proinflammatory and antiapoptotic genes (214, 215). Although high doses of D-gal inhibite the synthesis of antiapoptotic genes by depleting uridine triphosphate in hepatocytes, they promote apoptosis signaling via activation of the caspase cascade and DNA damage. Thus, TNF-α-mediated apoptotic signaling and inflammation are commonly considered pathophysiological mechanisms of D-Gal/LPS-induced ALF. Notably, the interaction between these mechanisms remains uncertain and should be explored in the future.

BMSC transplantation rescued the D-gal-induced liver failure model. In rodents, the 4-week survival rate significantly increased by 80% (216). Numerous hepatocytes were repaired, with only a few necrotic areas. In D-gal-induced liver failure in large animals, BMSC therapy significantly prolonged the survival time from 3.22 days to more than 14 days by suppressing the life-threatening cytokine storm. BMSC-derived hepatocytes were widely distributed in injured livers within 10 weeks, with liver function returning to normal levels (217). During recovery, serum levels of proinflammatory molecules, including IFN-γ, IL-1β, and IL-6, were reduced, while serum levels of the anti-inflammatory cytokine IL-10 were significantly increased through paracrine effects, referring to regulation by the STAT3 signaling pathway (216) and notch-DLL4 signaling pathway (218).

### 6.5 JO2-induced Fas/FasL-mediated liver failure model

Fas receptor (CD95), a member of the TNF-receptor superfamily with a death domain, mediates the assembly of a death-inducing signaling complex. Inducing caspase activation and cell apoptosis (219) has been considered the critical mechanism of fulminant liver failure (220), ischaemia-reperfusion-associated liver diseases (221), nonalcoholic fatty liver disorders (222) and other acute and chronic hepatic disorders. The liver constitutively and abundantly expresses the Fas receptor and activated caspase (casp) 8 upon binding of FasL or other receptor agonists (220, 223), such as the Fas receptor antibody JO2 and soluble FasL in the hexameric form (MegaFasL) (198). Then, it triggers the caspase cascade accompanied by excessive hepatocyte apoptosis (224), which can quickly progress to secondary necrosis (225). This mechanism may rely on activation of Fas-induced inflammatory signaling via the nonclassical interleukin-1β pathway (225).

Several researchers have reported that JO2 at concentrations such as 0.15, 0.2, 0.23, 0.35, 0.4, 0.42, and 0.5 mg/kg can induce liver failure, and the severity of liver injury is dependent on the JO2 dose (220, 221, 225–231). Thus, the critical element for developing this animal model is the concentration of JO2. Shao et al. (220) observed severe liver damage, including destruction of the hepatic lobules, hepatocyte necrosis and haemorrhage after treatment with 0.5 mg/kg JO2 (dissolved in normal saline (NS)) in BALB/c mice via intraperitoneal injection. However, after treatment of C57BL/6 mice with the same doses and methods, all mice died within 12 h (231). Although this difference could be caused by differences across researchers or other environmental factors, strain differences. In addition, the administration route can affect the pharmacodynamics and pharmacokinetics of the drug.

In recent works, BMSC transplantation rescued mice with JO2-induced liver failure and prolonged the survival time by improving liver function and decreasing extensive hepatic necrosis and haemorrhage. BMSCs can colonize injured mice and transdifferentiate into hepatocytes and cholangiocytes, and many KRT7- and KRT19-positive human cholangiocytes form tubular structures around the portal area (22). Meanwhile, transplanted BMSCs differentiate into immune cell lineages, including T cells, B cells, natural killer (NK) cells, macrophages and dendritic cells, and play a paracrine role by regulating inflammatory cytokine levels (23). Another study confirmed this finding and further identified the two transdifferentiation phases by transcriptomics; hepatic metabolism and liver regeneration were characterized in the first 5 days after BMSC transplantation, and immune cell growth and extracellular matrix (ECM) regulation were observed from day 5 to day 14 (25).

## 7 BMSC transplantation routes in animal and clinical trials

In clinical trials, MSC transplantation routes involving intravenous injection, followed by intrahepatic injection (through the portal vein and hepatic artery), and intrasplenic injection are minimally used (232). Notably, different routes could affect the number of MSCs homing to sites of damage. Next, we discuss which routes resulted in optimal therapeutic benefits.

Peripheral intravenous injection, such as caudal or jugular venous injection, is the most common administration route in clinical trials and animal models owing to the simplicity of the technique and the success rate rather than promising therapeutic results. In our previous study, we transplanted human-derived BMSCs (hBMSCs) into pigs with D-gal-induced fulminant hepatic failure (FHF) via peripheral and intraportal veins. The results revealed that all animals died of FHF within 96 hours after peripheral intravenous injection of hBMSCs, while most animals were rescued and survived for up to 6 months after intraportal vein injection (217), which suggested that the intraportal route has a better therapeutic effect than peripheral intravenous injection. E. Eggenhofer et al. radiolabelled MSCs with Cr-51 and found that within the first 24 hours after tail vein infusion, most viable MSCs accumulated in the lungs and that beyond 24 hours, MSCs disappeared in the lungs and were probably cleared by immune cells, with less than 10% of the cellular debris transferred to the injured liver (233). Similarly, Mami Higashimoto also found that a large proportion of MSCs resided in the lungs after caudal vein infusion, with only a small number of cells homing to the hepatic site of conA injury (234). This finding indicates that after the intravenous injection of MSCs, the cells first moved into the lungs and subsequently moved towards the liver, where they may be phagocytosed by reticuloendothelial cells in the capillary tissue, diminishing their therapeutic potential.

In contrast to intravenous injection, the intrahepatic portal vein is an important structure in the hepatic portal system that allows MSCs to rapidly home and colonize the liver after grafting and avoids cellular off-target effects. A comprehensive preclinical study compared four different transplantation routes: intraportal injection, intrahepatic artery injection, intravenous injection and intrahepatic injection. The results indicated that compared to other routes, intraportal injection of MSCs efficiently improved liver function, inhibited apoptosis and prolonged survival in ALF swine (235). An additional study confirmed that portal vein grafts can reduce the inflammatory response, inhibit cellular necrosis and promote liver regeneration in pigs with ALF (236). This preclinical evidence can ultimately guide the choice of graft route for the treatment of MSCs in the clinic. Regarding whether portal injection performs better in clinical trials, there was a trial comparing the therapeutic efficacy of MSCs after portal vein and intrasplenic injection in patients with end-stage liver diseases. According to the Fatigue Impact Scale and the MELD score, portal injection was found to be more effective than intrasplenic injection only in the first month, and this difference disappeared in the following months. The results demonstrated that the portal vein is more beneficial for the migration of MSCs. In particular, splenic injection could be the most promising route of transplantation in the future because of its simplicity (237). Recently, Ogasawara et al. found that the limited transplantable space in the spleen resulted in many cells clustered together experiencing high pressure, which may inhibit graft function (238). However, another animal study showed that transplantation of BMSCs via intrasplenic injection rescued a large proportion of 84.6% of FHF mice (23).

It is clear that intraportal injection can be chosen as the optimal administration route for MSCs to treat liver failure. Another possible reason for the excellent performance of portal vein transplantation is that there is an adequate graft area, and the graft can be widely distributed within the hepatic sinusoids and be maintained in good condition (238).

## 8 Future challenges and perspectives

MSCs are recognized as a promising cell therapy for the treatment of complications of liver transplantation, liver cancer, cirrhosis and liver failure caused by HBV, HCV, alcohol, primary biliary cholangitis and other infections (17, 18). Autologous bone marrow MSCs are the predominant source of cells, but the aspiration of bone marrow from patients themselves is still an invasive procedure, and the therapeutic efficiency of bone marrow MSCs can be limited by cellular senescence and differential proliferation and differentiation capacity (239). Adipose-derived MSCs may be an alternative source of cells in the future with the improvement of complex isolation strategies, and umbilical cord-derived MSCs would be a more desirable source of cells without the limitations mentioned above (240). While embryonic stem cell-derived extracellular vesicles have been verified to rejuvenate senescent MSCs and enhance their therapeutic effects, the antisenescence mechanism may be associated with the IGF1/PI3K/AKT pathway (241). MSC-derived extracellular vesicles have also been explored as a cell-free therapy that can effectively treat liver failure and avoid cell rejection (242–245).

The persistence time of MSCs for continued remission and maintenance of liver function have no consistent conclusion in various studies. Some clinical trials for acute-on-chronic liver failure showed that the MSC group can significantly improve liver function at 24 weeks or 48 weeks of follow-up, as shown in **Table 2**, alleviate TBIL levels and MELD scores, and decrease the mortality rate (247, 248, 251–253, 255). However, some studies have found that MSC transplantation has no significant effect (249, 256, 257). These results may be caused by the source of MSCs, quantity of MSCs, cell dosage, treatment frequency, endpoints and small number of cohorts.

**Table 2:** Clinical trials of MSC therapy in acute liver disease.

Lver disease	Cell type	Cell dosage	Time of treatment	Treatment interval	Administration route	Follow-up time	Improvement of liver function	Adverse event	Author
Acute-on-chronic liver failure	Autologous BMSCs	(3.4 ± 3.8) *10^8	1	/	Hepatic arterial injection	192 weeks	Levels of ALB, TBIL, and PT and MELD score were significantly improved from 2-3 weeks after transplantation in MSC group	No serious side effects or complications;	Liang Peng, Zhi-liang Gao et al. (246)
Acute-on-chronic liver failure	Allogenic UMSCs	1*10^6/kg	1	/	Intravenous injection	24 week or 72 weeks	1. The mortality rate was significantly decreased (MSC group vs. control, 20.8% % v.s. 47.7% at 72 weeks of follow-up 2. MSC therapy significantly reduced MELD score, increased ALB, cholinesterase, prothrombin activity and platelet counts, decreased TBIL ALT and AST level at 24-week of follow-up	No significant side effects	Ming Shi, Fu-Sheng Wang et al. (247)
hepatitis B chronic with decompensated Liver cirrhosis	Allogenic UMSCs	0.5*10^6/kg	3	Every 4 weeks	Intravenous injection	48 weeks	1. A significant reduction of volume of ascites in MSC group 2. Liver function significantly improved revealed by the increased of ALB level, and the decreased TBIL level and MELD score	No significant side-effects and complications	Fu-Sheng Wang et al. (248)
Decompensated cirrhosis	Autologous BMSCs	1.20-2.95*10^8	1	/	Peripheral vein	48 weeks	Child scores, MELD scores, ALB, INR, ALT, AST and liver volumes have no significant effect in MSC group at 48-week of follow-up	Fever	Mehdi Mohamadnejad et al. (249)
HCV-related decompensated cirrhosis	Autologous BMSCs	1*10^6/kg	1	/	Intrahepatic infusion	24 weeks	MSC therapy improved ALB within the first 2 weeks and prothrombin concentration and ALT after 1 month	No	Hosny Salama et al. (250)
Hepatitis B chronic plus acute liver failure	Plasma exchange and Allogenic UMSCs	1*10^8/60 ml saline	1	/	Hepatic arterial injection	96 weeks	1. Cumulated survival rate at 24 weeks was significantly improved (MSC group vs. control, 54.5% % v.s. 26.5%) 2. The level of ALB, AST, ALT, TBIL, PT, INR and MELD score significantly improved at 24 weeks	No severe adverse event	Yu-Hua Li et al. (251)
Acute-on-chronic liver failure	Allogenic UMSCs	(1.0-10) *10^5/kg	4	Every 1 week	Intravenous injection	24 weeks	1. Cumulated survival rate for follow-up 24 weeks was significantly improved (MSC group vs. control, 73.2% v.s. 55.6%) 2. The laboratory indexes, including alanine aminotransferase (ALT) and albumin (ALB) had significantly improved in MSC group at week 1, and MELD score dramatically decreased at week1 and 2 3. The incidence of severe infection in the MSC group was much lower	No infusion-related side effects, No carcinoma event, Fever	Bing-liang Lin et al. (252)
Acute-on-chronic liver failure	Plasma exchange and Allogenic UMSCs	1*10^5/kg	4	Every 1 week	Intravenous injection	48 weeks	1. Rates of death and unfavourable outcome were decreased without significances. 2. TBIL, ALT, AST and MELD score were significantly decreased during treatments	Fever	Wen-xiong Xu et al. (253)
Acute-on-chronic liver failure	Allogenic BMSCs	1*10^6/kg	5	Twice in the first and second weeks, and once in the third week.	Intravenous injection	12 weeks	1. Survival rate after 12-week follow-up (MSC group vs. control, 25% vs. 20%) 2. MSC therapy significantly improved Child–Pugh score (C-14 to B-9), MELD score (32 to 22) and ACLF grade (3 to 0)	No infusion-related side effects	Fernando Comunello Schacher et al. (254)
Decompensated liver cirrhosis	Allogenic UMSCs	0.5*10^6/kg	3	Every 4 weeks	Intravenous injection	300 weeks	1. Follow-up period 13 to 75th months, MSC group significantly prolonged overall survival rate 2. Liver function (including ALB, prothrombin activity, cholinesterase, TBIL) markedly improved during 48 weeks of follow-up	No Significant side effect; No hepatocellular carcinoma event	Ming Shi et al. (255)

BMSCs, bone marrow mesenchymal stem cells; UMSCs, umbilical cord mesenchymal stem cells; ALB, albumin; TBIL, total bilirubin; ALT, alanine aminotransferase; AST, aspartate aminotransferase; MELD score, Model for End-stage Liver Disease score.

The quality of MSCs is a critical factor that needs to be considered, as in many clinical trials, the characteristics of autologous BMSCs from patients of different ages and disease states vary significantly; therefore, there is an urgent need to establish uniform criteria for evaluating the quality of MSCs to support autologous or allogeneic transplantation.

Although MSCs can improve liver function and effectively treat liver failure in the short term and can be used as a cell source to modulate cellular properties and improve the effectiveness of bioartificial liver systems, their long-term efficacy in patients with decompensated end-stage liver disease remains poor (246). Zhang Z et al. first reported 45 patients with chronic hepatitis B decompensation who received MSC transfusions at 0.5 x 10^6^ cells/kg three times at 4-week intervals. Clinical parameters were measured at 40 weekly follow-ups, and the results demonstrated that MSC treatment markedly reduced ascites and improved liver function in patients with decompensated liver cirrhosis (248). Other similar research also reported the effectiveness of multiple injections. Peripheral intravenous infusion of MSCs at a dose of 0.5 x 10^6^ cells/kg 3 times, 4 weeks apart for chronic hepatic failure and chronic hepatitis B liver failure, effectively prolonged the overall survival time and improved the biochemical liver index (247, 255, 258). A total of 0.5 x 10^7^ cells were injected via the hepatic artery twice in weeks 4 and 8 for alcoholic cirrhosis disease, which improved the patients’ liver histological features (259, 260). Multiple injections of MSCs may achieve long-term therapy. The treatment interval of MSCs that can maintain liver function is shown in **Table 2**, including every week for 4 weeks, twice in the first and second weeks and once in the third week for a total of 5 times, every four weeks for a total of three times and only one infusion (247, 252–255). However, no uniform guideline has been defined, and there is an urgent need to address this issue through extensive animal and clinical trials.

Current studies on MSC therapy have some limitations. MSCs are a heterogeneous population that limits their consistent treatment effects. Although **Table 1** lists the special markers of MSCs, cell subsets with specific biological functions have not been identified. The MSC atlas is an urgent acquirement for screening special cell subpopulations aimed at different diseases. The guidelines for isolating and cultivating high-quality MSCs have not been uniformed. The mechanisms of MSC therapy for acute liver failure are still poorly understood. How does MSC migrate to the site of injury from spatial distribution, and in what form does it treat hepatocyte failure and regulate the immune microenvironment. Therefore, multi-omics combinations, including spatial transcriptomics, single-cell transcriptomics, proteomics, metabolomics, and bulk transcriptomics, are an instant demand to generally clarify the mechanisms of MSC therapy. Highly simulated mouse models of human acute liver failure need to be constructed to better evaluate the efficacy of MSCs in preclinical studies and provide more evidence-based medical evidence for clinical trials. MSCs have been used in perioperative care for liver transplantation and to improve immune rejection after liver transplantation (10, 11). However, the persistence, frequency and early initiation time of MSC treatment have no consistent conclusion, which requires further validation in multi-central, large sample, non-random cohorts. The comparison and combination of MSC therapy and other strategies, such as xenotransplantation, is an important direction for future studies.

## Author contributions

HY and JC contributed equally. HY drafted the work. JL conceived and revised the work. JC drew and revised the figure. All authors contributed to the article and approved the submitted version.
